# Paraventricular thalamic neurons respond to vagus nerve activation and modulate anxious behavior

**DOI:** 10.3389/fncir.2026.1865790

**Published:** 2026-08-24

**Authors:** Ziyan Xie, Musashi Yamakawa, Tasuku Kayama, Takuya Sasaki, Nahoko Kuga

**Affiliations:** 1 Department of Pharmacology, Graduate School of Pharmaceutical Sciences, Tohoku University, Sendai, Japan; 2 Department of Neuropharmacology, Graduate School of Medicine, Tohoku University, Sendai, Japan; 3 Creative Interdisciplinary Research Division, Frontier Research Institute for Interdisciplinary Sciences, Tohoku University, Sendai, Japan

**Keywords:** anxiety, interoception, paraventricular thalamic nucleus (PVT), spike, vagus nerve

## Abstract

Interoceptive signals reflecting internal bodily states influence emotional processing. The paraventricular thalamus (PVT) constitutes a key node within the interoceptive network, monitoring circulating humoral factors in the ventricles, and is thus considered to link interoceptive signals to emotion-related behaviors. Here, we demonstrate that the vagus nerve (VN), a principal afferent pathway for interoceptive signaling, also affects PVT neuronal activity in mice. Left vagus nerve stimulation (VNS) induced pronounced c-Fos expression in PVT neurons and elicited transient increases and decreases in neuronal spike rates across all PVT subregions. These VNS-responsive neuronal populations were distinct from those exhibiting transient-to-sustained delayed responses following lipopolysaccharide injection after vagotomy, which selectively activates VN-independent pathways, including humoral factors. VNS-responsive PVT neurons projected to limbic structures and modulated anxiety-like behavior. Taken together, these findings demonstrate that PVT neuronal populations integrate and relay interoceptive signals across multiple pathways and timescales, thereby playing a pivotal role in linking vagus nerve-related bodily states to emotional responses.

## Introduction

The brain receives diverse interoceptive signals that reflect internal states of the body, which are critical for emotional processing (Critchley and Garfinkel, 2017; Khalsa et al., 2018). The paraventricular thalamus (PVT) is a key node of the interoceptive network, positioned adjacent to the dorsal third ventricle with direct access to the ventricular environment reflecting the state of circulating humoral factors. PVT neurons project to diverse limbic and cortical areas, positioning the PVT as a key region for emotional regulation (Kurokawa et al., 1977; Hsu et al., 2014; Barson et al., 2020; McGinty and Otis, 2020; McNally, 2021). These anatomical features suggest that the PVT serves as a potential relay region linking interoceptive signals to emotion-related and motivated behavior (Meffre et al., 2019; Machen et al., 2026).

Furthermore, the PVT also receives projections from brainstem and hypothalamic regions that process interoceptive information conveyed via peripheral nerves. One major nerve pathway is the afferent vagus nerve (VN) (Critchley and Garfinkel, 2017; Quadt et al., 2018; Berntson and Khalsa, 2021; Chen et al., 2021), which monitors dynamic physiological changes, including visceral organ activity (e.g., heartbeat, digestion) and peripheral hormonal release (e.g., insulin) (Berthoud, 2008; Iwasaki and Yada, 2012; Prescott and Liberles, 2022; Masuda et al., 2025; Mendez-Hernandez et al., 2025), and has been implicated in emotional regulation (Nemeroff et al., 2006; Bravo et al., 2011; Klarer et al., 2014; Conway et al., 2025). Taken together, the PVT may also be modulated by interoceptive information via the afferent VN, in addition to humoral factors via the ventricles, suggesting that it integrates these inputs to control emotional behavior.

**Figure 1:**
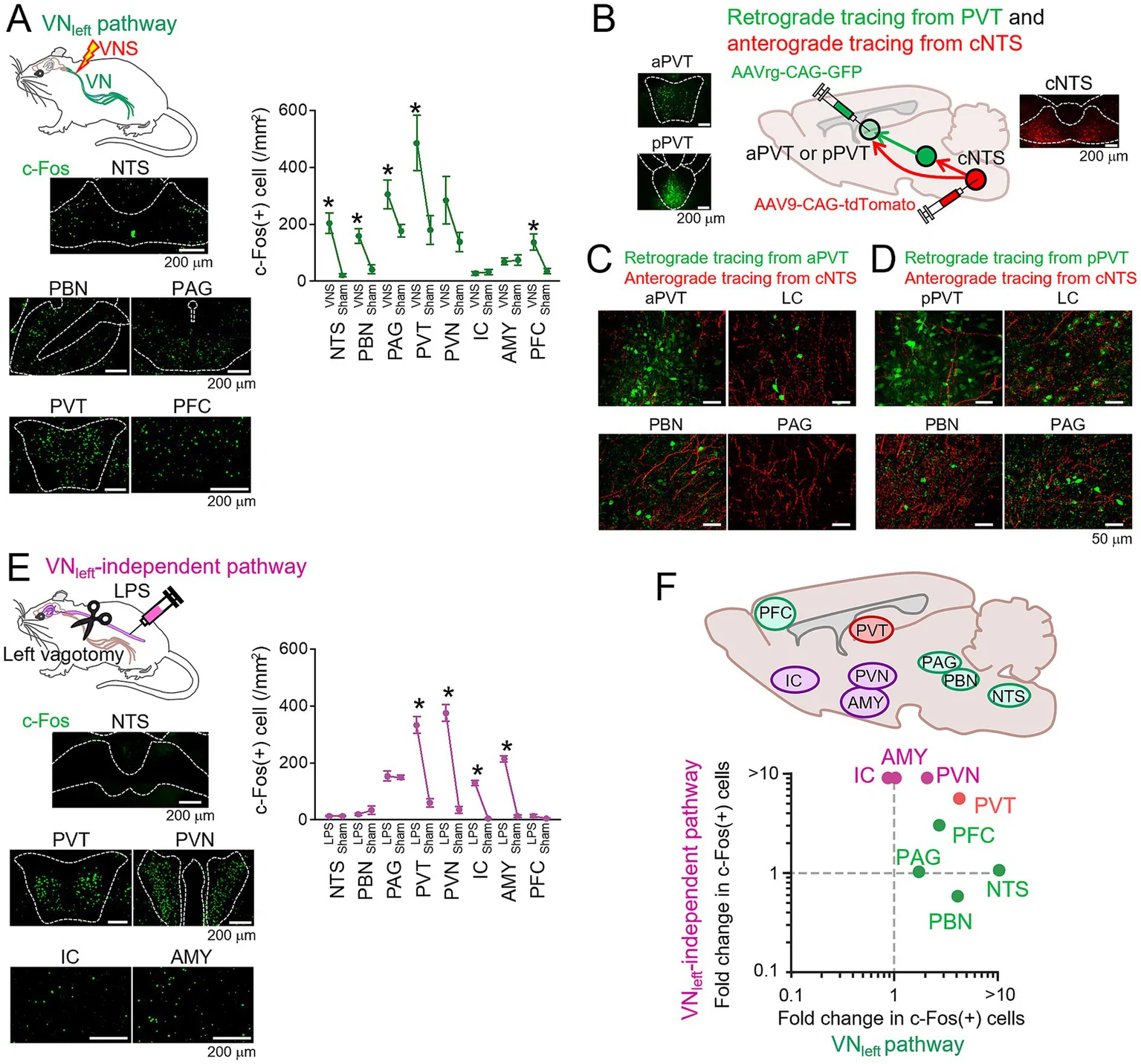
Activation of PVT neurons by the VN_left_ and VN_left_-independent pathways. **(A)** The VN_left_ was activated by VNS. (Left) Representative pictures of c-Fos-expressing neurons. (Right) The density of c-Fos-positive neurons, compared with sham controls (*n* = 7–9 sham and 7–9 VNS mice). Error bars represent SEM. *p < 0.05, Student’s *t*-test. **(B)** AAV9-CAG-tdTomato was injected into the cNTS, and AAVrg-CAG-GFP was injected into the aPVT or pPVT. Fluorescent images confirming GFP expression in aPVT or pPVT neurons (left) and tdTomato expression in cNTS neurons (right). **(C)** Fluorescent images of tdTomato-labeled cNTS axonal fibers (red) and retrogradely GFP-labeled neurons projecting to the aPVT (green) in each brain region. **(D)** Same as C, but showing neurons projecting to the pPVT. **(E)** The VN_left_-independent pathway was activated by LPS administration following left vagotomy. (Left) Representative pictures of c-Fos-expressing neurons. (Right) c-Fos-expressing neurons were analyzed, compared with sham controls (*n* = 6–7 sham and 6–10 LPS-treated mice). Error bars represent SEM. *p < 0.05, Student’s *t*-test. **(F)** (Top) Summary of brain regions activated by the VN_left_ (green) and VN_left_-independent (purple) pathways. The PVT was activated by both pathways (red). (Bottom) Relationship between fold changes relative to sham controls in c-Fos-positive neurons in each brain region in the VN_left_ (*x-*axis) and VN_left_-independent pathway (*y*-axis).

To investigate the relationship between PVT neurons and the VN, we applied vagus nerve stimulation (VNS) in mice and found that PVT neurons showed increased c-Fos expression in response to VNS (Figure 1), suggesting that PVT neurons are substantially influenced by vagal input. An important open question is how VN-related PVT neurons encode interoceptive signals at the level of individual spikes and on specific timescales, and whether they integrate or segregate inputs from other pathways such as humoral signals. At the behavioral level, it is also critical to determine whether VN-responsive PVT neuronal ensembles contribute to emotional regulation.

To address these questions, we performed *in vivo* spike recordings to characterize the temporal response patterns of individual PVT neurons to VN-dependent and -independent interoceptive signals, and examined the role of VN-responsive neuronal ensembles in emotional behavior. Here, we focused on the left VN (VN_left_), as the left side has been reported to be primarily involved in anxiolytic and emotion-related functions (Okonogi et al., 2024), and left VNS has been widely employed in the treatment of various neurological and psychiatric disorders (Howland, 2014; Austelle et al., 2022). To more precisely delineate the functional organization of the PVT, we analyzed its physiological and behavioral characteristics by subdividing it into the anterior, central, and posterior PVT (aPVT, cPVT, and pPVT), as these subregions have been reported to exhibit distinct projection patterns and functional roles (Barson et al., 2020; Iglesias and Flagel, 2021).

## Materials and methods

### Approvals

All experiments were performed with the approval of the animal experimentation ethics committee at Tohoku University. (Approval number: 2022 PhA-004) and in accordance with the NIH guidelines for the care and use of animals.

### Subjects

Male C57BL/6 J mice (8–10 weeks old, 22–27 g) and male ICR mice (3–6 weeks old, 18–40 g) were used. All wild-type mice were obtained from SLC (Shizuoka, Japan). TRAP2 mice (8–10 weeks old, 29–35 g; B6.129(Cg)-*Fos^t2.1(cre/ERT2)Luo^*/J; stock #021882, Jackson Laboratory) were also used. Results from only male mice were reported in this study because the effects of VNS on brain activity and behavior were not fully reproducible in females. Mice were housed under controlled conditions (23 ± 1 °C; 50 ± 5% relative humidity) with a 12-h light/12-h dark cycle (lights off at 8:00 p.m.) and had ad libitum access to food and water.

### Surgery for vagus nerve manipulation and drug/virus injections

Mice were anesthetized with 3% isoflurane gas and then maintained with 1–3% isoflurane gas while lying on their backs. Veterinary ointment was applied to the eyes of each mouse to prevent dryness. Prior to each incision, the skin was sterilized with betadine followed by 70% ethanol.

For vagotomy and VNS, an incision was made in the neck area from the larynx to the sternum, and the bundle containing the VN_left_ and the carotid artery was isolated. The cervical VN_left_ was further isolated from the surrounding tissue and the carotid artery. For vagotomy, the cervical VN_left_ was transected with surgical micro-scissors. For VNS, the isolated cervical VN_left_ was enclosed by a custom-made cuff-shaped VN electrode, with the electrode contact sites attached to the inner wall of the cylindrical tube (inner diameter = 0.5 mm, electrode area = 0.15 mm^2^, cathode–anode interval = 0.5 mm, total length = 2.0 mm) (Kuga et al., 2019; Mughrabi et al., 2021; Okonogi et al., 2024). We targeted the VN_left_ because (1) compared with the right VN, the VN_left_ has fewer connections to the sinoatrial node, and therefore stimulation of VN_left_ induces fewer adverse effects on cardiac function in both humans and rodents (Bagot et al., 2016), and (2) VN_left_ has been reported to exert anxiolytic effects and to modulate emotion-related functions (Okonogi et al., 2024).

For tracing the neuronal pathway from the cNTS to the PVT, AAV9-CAG-tdTomato (UNC Vector Core) dissolved in phosphate-buffered saline (PBS; pH 7.4) was injected into the cNTS using a glass pipette (*ϕ* = 10 μm). The cNTS was targeted at the following coordinates (mm from obex) (AP, −0.5; ML, ±0.2; DV, −0.5) and the virus was infused at a rate of 50–100 nL/min for 1 min using a syringe pump (Muromachi Kikai, Tokyo, Japan). In the same animals, AAVrg-CAG-GFP (UNC Vector Core) dissolved in PBS was injected into either the aPVT (AP, −0.4; ML, 0.0; DV, −3.4) or the pPVT (AP, −1.8; ML, 0.0; DV, −3.3) (mm from bregma) at a rate of 50–100 nL/min for 3 min. After each injection, the pipette was left in place for 1 min, raised by 50 μm, and then left in place for an additional 5 min before withdrawal.

For mapping and chemogenetic manipulation of neurons labeled using the TRAP2 system, either AAV9-CAG-FLEX-eGFP, AAV8-hSyn-DIO-mCherry, or AAV8-hSyn-DIO-hM4Di-mCherry (UNC Vector Core) was injected into the PVT. Stereotaxic coordinates (mm from bregma) were as follows: aPVT (AP, −0.4; ML, 0.0; DV, −3.4), cPVT (AP, −1.4; ML, 0.0; DV, −3.3), or pPVT (AP, −1.8; ML, 0.0; DV, −3.3). Viruses were infused at a rate of 50–100 nL/min for 3 min, as described above.

Following surgery, each animal was housed individually in a transparent Plexiglas cage with free access to water and food.

### VNS procedure

To apply VNS to the mice, the socket was connected to an electrical stimulator (Unique Medical) via a wire. Stimulation (0.8 mA, 0.1 ms pulse width, 20 Hz) was delivered in cycles of 0.5 s on and 9.5 s off for 10 min during electrophysiological recordings. For c-Fos immunostaining and activity-dependent labeling in TRAP2 mice, stimulation was delivered in cycles of 30 s on and 30 s off for 3 h. For c-Fos immunostaining, the mice were transcardially perfused 90 min after VNS.

### Lipopolysaccharide (LPS) administration

Mice were injected intraperitoneally with lipopolysaccharide (LPS; *Escherichia coli* K-235, Sigma-Aldrich) at a dose of 1 mg/kg. For c-Fos immunostaining, mice were transcardially perfused 3.5 h after LPS injection.

### Chemogenetic manipulations

To label VN_left_-responsive PVT neurons in TRAP2 mice, 4-hydroxytamoxifen (4-OHT; 50 mg/kg; Sigma-Aldrich; dissolved in a mixture of ethanol and sunflower oil, 1:19 v/v, at a final concentration of 5 mg/mL) was administered intraperitoneally to induce CreER-mediated recombination in c-Fos-expressing neurons. For subsequent chemogenetic inhibition of VN_left_-responsive PVT neurons, water-soluble deschloroclozapine (DCZ; 0.1 mg/mL; Tocris Bioscience) was administered via drinking water for up to 2 weeks. Mice in which viral expression was not confirmed within the target subregion were excluded from subsequent analyses.

### Electrophysiological recording

For electrophysiological recording, the mice were anesthetized with urethane (1.0 g/kg, intraperitoneal injection) and restrained with their head held in place by a metal plate (Nishimura et al., 2016; Nishimura et al., 2021a, 2021b). A craniotomy was performed using a high-speed drill to create a hole (1.0 × 1.0 mm^2^) centered at the following coordinates relative to the bregma: −0.4 mm anterior–posterior (AP), 0.0 mm lateral (aPVT); −1.5 mm AP, 0.0 mm lateral (cPVT); and −1.9 mm AP, 0.0 mm lateral (pPVT), and the dura was surgically removed. A stainless-steel screw was implanted in the bone beside the hole to serve as ground and reference electrodes. A silicon probe that consisted of 64 recording sites (Buzsáki64, NeuroNexus) was inserted into the exposed area at a speed of 10 μm/s. The final depth of the probe in the brain ranged from 3,100 to 3,500 μm below the cortical surface. The probe was allowed to stabilize at its final position for 30 min before recording began. To aid in reconstructing electrode tracks, probes were coated with the fluorescent dye DiI by dipping the electrode tips into a DiI solution (Invitrogen; 80 mg/mL, dissolved in 1:1 acetone/ethanol) for 60 s prior to insertion. Recordings began once stable neuronal unit activity was observed. For recording electrophysiological signals, the probe was connected to a Cereplex M digital headstage (Blackrock Microsystems), and the digitized signals were transferred to a Cereplex Direct data acquisition system (Blackrock Microsystems). Electrical signals were sampled at 2 kHz and low-pass filtered at 500 Hz. The unit activity was amplified and bandpass filtered at 750 Hz to 6 kHz. Spike waveforms above a trigger threshold (50 μV) were time-stamped and recorded at 30 kHz in a time window of 1.6 ms. At the end of the recording, the probes stained with DiI were carefully removed from the brain, unless otherwise specified. Recordings were performed at room temperature (22–24 °C).

After the recordings, the mice were intracardially perfused with cold 4% paraformaldehyde (PFA) in 25 mM phosphate-buffered saline (PBS) and subsequently decapitated for histological analysis to confirm electrode placement. After dissection, the brains were fixed overnight in 4% PFA and then equilibrated with a sequence of 20% sucrose and 30% sucrose in PBS. Frozen coronal sections (50 μm) were obtained, and serial sections were rinsed in water, and coverslipped with Permount. Electrode tracks were reconstructed based on DiI labeling to verify probe placement. For data analysis, only recording channels located within the PVT were included. Among the eight shanks of the silicon probe, shanks passing through the PVT were identified, and within these shanks, only channels unequivocally confined to the PVT were selected for subsequent analyses.

### Immunohistological analysis

The mice were overdosed with isoflurane, transcardially perfused with 4% PFA in PBS, and decapitated. After dissection, the brains were fixed overnight in 4% PFA and then equilibrated in 30% sucrose in PBS for an additional night. Frozen coronal sections (50 μm) were cut using a microtome.

For c-Fos, mCherry, and GFP immunostaining, brain slices were permeabilized in PBS containing 0.3% Triton X-100 and 5% bovine serum albumin (BSA) at room temperature for 60 min. Slices were then incubated overnight at 4 °C with the following primary antibodies: mouse anti-c-Fos (1:1000; Encor Biotechnology, MCA-2H2), rabbit anti-DsRed (1:1000; Takara Bio, 632,496), and chicken anti-GFP (1:1000; Abcam, ab13970). After rinsing with PBS, slices were incubated for 90 min at room temperature with Alexa Fluor 488-conjugated anti-mouse IgG (1:1000), Alexa Fluor 594-conjugated anti-rabbit IgG (1:1000), and Alexa Fluor 488-conjugated anti-chicken IgG (1:1000) (Jackson ImmunoResearch) in PBS containing 0.3% Triton X-100. Samples were mounted with Fluoro-KEEPER Antifade Reagent (Nacalai Tesque). Images were acquired using a fluorescence microscope (BZ-X800; Keyence, Osaka, Japan) equipped with a water-immersion objective lens (×10, 0.75 NA). c-Fos–positive cells were quantified by counting cells exceeding a defined fluorescence intensity threshold within each region of interest (ROI). Axonal projections were quantified as projection density, calculated as the proportion of GFP-positive pixels within each ROI. All analyses were performed using analysis software (BZ-H4C; Keyence, Osaka, Japan).

### Elevated plus maze test

An EPM consisted of a central square (7.6 × 7.6 cm) connected to four arms (28 cm long × 7.6 cm wide): two open arms without railings and two closed arms enclosed by 15-cm-high walls. The maze was elevated 70 cm above the floor. During testing, each mouse was placed in the central square facing an open arm and allowed to explore the maze for 20 min. For behavioral analyses, the first 15 min of exploration were analyzed. The time spent in the open and closed arms was measured, and video recordings were downsampled to 3 Hz for analysis.

### Open field test

An open field consisted of a 42 × 42 cm area enclosed by 42-cm-high walls. During testing, each mouse was placed in the center zone and allowed to explore the arena for 15 min. The time spent in the center zone was measured, and video recordings were downsampled to 3 Hz for analysis.

### Food intake test

Each mouse had free access to standard chow in its home cage throughout the chemogenetic manipulation period. Food intake was assessed by weighing the remaining chow daily over 5 days.

### Spike analysis

Each shank of the Buzsaki64 silicon probe contains 8 recording channels. We selected 4 adjacent channels with high-quality signals as a multiunit cluster and analyzed them using the same approach as tetrode recordings. Spike sorting was performed offline using the graphical cluster-cutting software MClust. All recording periods were included in the analysis to confirm recording stability throughout the experiment. Clustering was performed manually in two-dimensional projections of the multidimensional parameter space (i.e., waveform amplitudes, waveform energies, and principal components of waveforms recorded across the four channels). Cluster quality was measured by computing the *L_ratio_* and the isolation distance (Supplementary Figure S2B). The *L_ratio_* was computed by the original equation, not normalized by the total number of spikes recorded on the tetrode. A cluster was considered as a cell when the *L_ratio_* was less than 0.30.

Spike rates were calculated for individual neurons using 1-min bins. Three periods were defined: Pre (0–20 min before LPS injection), Post 1 (0–20 min after LPS injection), and Post 2 (30–50 min after LPS injection). To assess changes in spike rates during Post 1 or Post 2 relative to Pre, statistical comparisons were performed between the 20 binned spike frequency values in each period using Student’s *t*-test (when data were normally distributed) or Mann–Whitney U test (when data were not normally distributed). Individual neurons were classified as transient, delayed, or sustained types depending on whether significant changes occurred in Post 1, Post 2, or both periods.

For each cell, the spike waveform was characterized by half-width and peak-trough ratio. Half-width was defined as the time interval between the two points at which the waveform crossed 50% of the peak-trough amplitude, measured relative to the primary negative deflection (trough) of the spike. Peak-trough ratio was calculated as the ratio of the voltage at the positive peak to the voltage at the trough. Burstiness was assessed from the interspike interval (ISI) distribution, calculated as the difference between the standard deviation and the mean of the ISI, divided by the sum of these two values: [std(ISI) − mean(ISI)]/[std(ISI) + mean(ISI)].

## Data analysis

All data were analyzed using MATLAB and are presented as mean ± SEM unless otherwise indicated. When variability is expressed as standard deviation (SD), this is noted in the figure legends. Two-sample comparisons were performed using Student’s *t*-test or paired *t*-test. When data were not normally distributed, the Mann–Whitney U test was applied. For multiple group comparisons, the Kruskal–Wallis test followed by Dunn’s *post hoc* test was used. For categorical data, chi-square tests with Bonferroni correction were applied for pairwise comparisons. Repeated-measures data were analyzed using two-way repeated-measures ANOVA with Greenhouse–Geisser correction. Correlations were assessed using Spearman’s correlation coefficient. For overlap analyses, the expected overlap by chance was calculated from the product of the activation probabilities of the VN_left_ and VN_left_-independent pathways and compared with the observed overlap using Fisher’s exact test. Statistical significance was set at *p* < 0.05.

## Results

### PVT neurons respond to VN_left_ activation

To identify neuronal populations responsive to VN signals, we analyzed c-Fos expression, an immediate early gene marker of neuronal activation, across brain regions implicated in the processing of interoceptive signals. The VN_left_ was stimulated via a cuff-shaped electrode that was chronically implanted on the VN_left_. VNS consisted of 600 pulses delivered every 1 min for 3 h (0.8 mA, 0.1 ms pulse width, 20 Hz) (Figure 1A). Compared with sham controls (electrodes implanted without stimulation), VNS-treated mice showed significantly higher proportions of c-Fos-positive neurons in the nucleus tractus solitarius (NTS), the primary target of afferent vagal inputs (Figure 1A; *n* = 9 sham and 9 VNS mice, *t_16_* = 5.09, *p* = 1.1 × 10^−4^, Student’s *t*-test), validating the efficacy of VNS. Under these conditions, significant increases in c-Fos-expressing neurons were observed in the parabrachial nucleus (PBN), periaqueductal gray (PAG), PVT, prefrontal cortex (PFC), including the prelimbic and infralimbic cortex (Figure 1A, *n* = 7–9 sham and 7–9 VNS mice; PBN: *t_14_* = 3.59, *p* = 0.0029; PAG: *t_16_* = 2.29, *p* = 0.036; PVT: *t_16_* = 2.79, *p* = 0.013; PFC: *t_15_* = 3.14, *p* = 0.0067, Student’s *t*-test), but not in the paraventricular nucleus of the hypothalamus (PVN), insular cortex (IC), including the anterior and posterior parts, and amygdala (AMY), including the central and basolateral parts (PVN: *t_14_* = 1.77, *p* = 0.098; IC: *t_16_* = 0.34, *p* = 0.74; AMY: *t_16_* = 0.22, *p* = 0.83, Student’s *t*-test).

We examined how VN_left_-induced signals are conveyed to the PVT. As the VN primarily terminates and synaptically connects to the caudal NTS (cNTS), we next examined whether cNTS neurons project directly to the PVT or reach the PVT via intermediate brain regions. To address this, we combined anterograde tracing from the cNTS using AAV9-CAG-tdTomato with retrograde tracing from the aPVT or pPVT using AAVrg-CAG-GFP (Figure 1B). TdTomato-positive axonal projections from the cNTS were observed in both the aPVT and pPVT (Figures 1C,D, red), confirming that the cNTS directly sends monosynaptic projections to the PVT. Furthermore, within the same preparations, cNTS-derived axonal projections and retrogradely labeled neurons targeting the aPVT and pPVT were co-observed in the locus coeruleus (LC) and PBN. Similarly, cNTS-derived axonal projections and retrogradely labeled neurons targeting the pPVT were observed in the PAG. Together, these anatomical findings suggest that VN-related signals are relayed to the PVT via these intermediate regions.

We next tested brain regions, including the PVT, activated by interoceptive pathways independent of the VN_left_ (VN_left_-independent pathway). These alternative pathways include the right VN, spinal sensory pathways, and humoral routes via the bloodstream. Because systemic LPS administration is known to activate all of these pathways, we used LPS as a tool to comprehensively engage VN_left_-independent interoceptive signaling. Mice underwent left vagotomy and were administered LPS (1 mg/kg, i.p.) (Figure 1E). Compared with sham controls (PBS injection), LPS injection significantly increased c-Fos-positive neurons in the PVT, PVN, IC, and AMY (*n* = 7–9 sham and 5–10 LPS-treated mice; PVT: *t_13_* = 7.10, *p* = 8.1 × 10^−6^; PVN: *t_14_* = 8.38, *p* = 8.0 × 10^−7^; IC: *t_10_* = 13.38, *p* = 1.0 × 10^−7^; AMY: *t_14_* = 13.73, *p* = 1.6 × 10^−9^, Student’s *t*-test), but not in the NTS, PBN, PAG, and PFC (NTS: *t_10_* = 0.25, *p* = 0.81; PBN: *t_10_* = 0.87, *p* = 0.40; PAG: *t_11_* = 0.24, *p* = 0.82; PFC: *t_10_* = 1.42, *p* = 0.19). Taken together, these results demonstrate that VN_left_ or VN_left_-independent pathways activated several brain regions with little overlap (Figure 1F), and the PVT was the only region that responded to both pathways. When LPS was administered with the VN_left_ pathway intact, whereby both the VN_left_ and VN_left_-independent pathways were considered to be activated (Supplementary Figure S1A), all regions except the PAG showed significant increases in the proportion of c-Fos-expressing neurons (*p* < 0.05, Student’s *t*-test), suggesting an additive effect of the two pathways.

### PVT neurons show transient changes in spike rates by VNS

**Figure 2:**
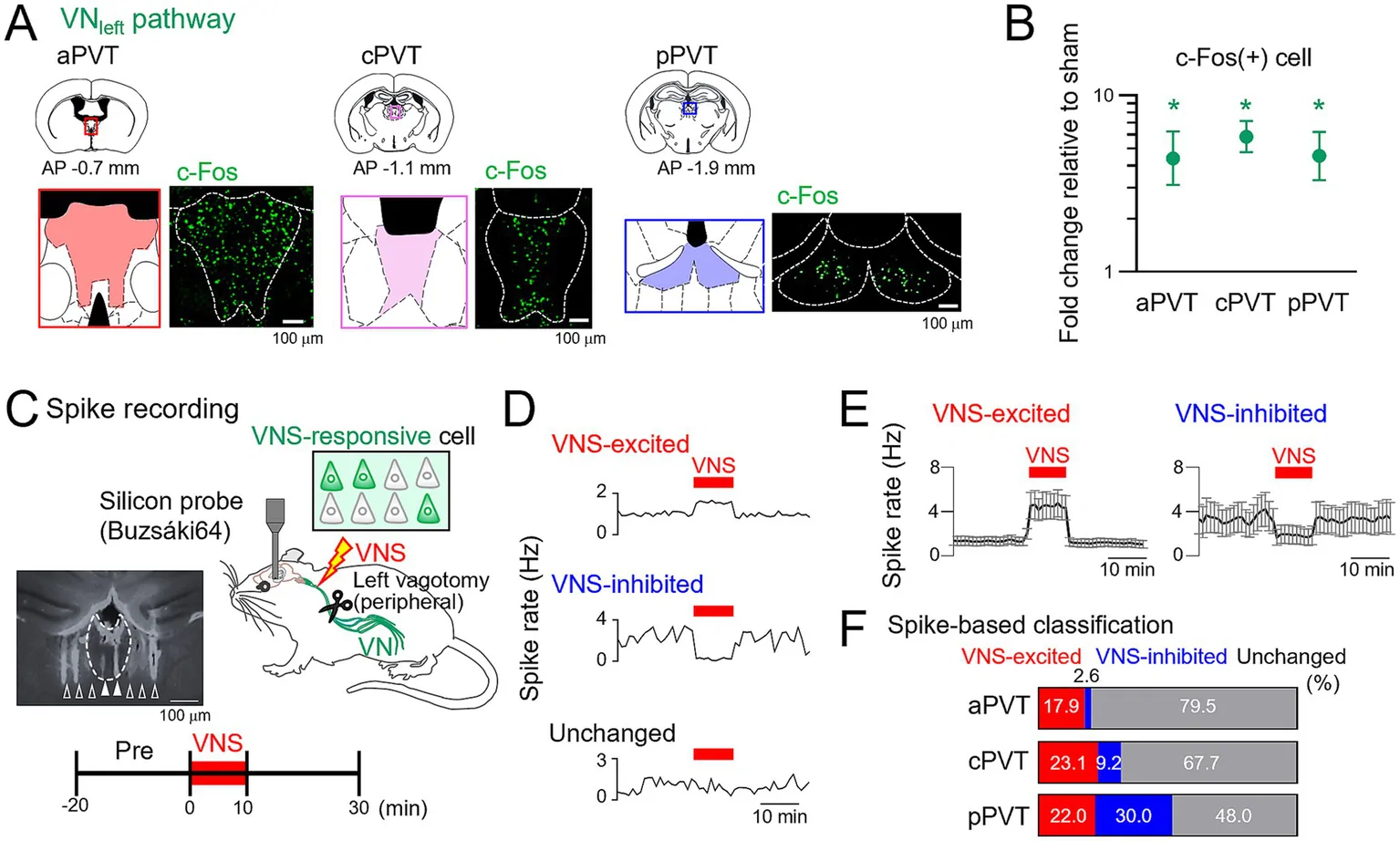
Spike responses of PVT neurons to VN_left_ pathway activation (VNS). **(A)** Pictures of c-Fos-expressing neurons in each PVT subregion after VNS. **(B)** Fold changes in c-Fos-positive neurons relative to sham controls (*n* = 9–11 sham and *n* = 7–8 VNS mice). Error bars represent SD. *p < 0.05, Mann–Whitney U test for aPVT and cPVT; Student’s *t*-test for pPVT. **(C)** Spike patterns were recorded from PVT neurons using eight-shank silicon probes in urethane-anesthetized mice. In the PVT histological picture, filled and open arrowheads represent electrodes located within and outside the PVT, respectively. Left vagotomy was performed peripheral to the stimulation site before recording, and VNS was applied for 10 min during recording. **(D)** Representative neurons showing increased (VNS-excited), decreased (VNS-inhibited), or unchanged spike rates in response to VNS. **(E)** Averaged absolute changes in spike rates from all VNS-excited or VNS-inhibited neurons (*n* = 33 and *n* = 22 cells). Error bars represent SEM. **(F)** Proportions of neurons in each PVT subregion (*n* = 39, *n* = 65, and *n* = 50 cells).

We next subdivided the PVT into three subregions and examined VNS-induced neuronal c-Fos expression: the aPVT (AP − 0.23 to −0.95 mm), cPVT (AP − 0.95 to −1.67 mm), and pPVT (AP − 1.67 to −2.27 mm) (Figure 2A). All three subregions showed significant increases in c-Fos expression in response to VNS (Figure 2B; aPVT: *t_16_* = 5.55, *p* = 4.4 × 10^−5^; cPVT: *t_17_* = 10.48, *p* = 7.7 × 10^−9^; pPVT: *t_15_* = 5.23, *p* = 1.0 × 10^−4^, Student’s *t*-test). To further examine detailed temporal dynamics of changes in neuronal spike rates, we next performed multiunit spike recordings from PVT neurons (Figure 2C). Under urethane anesthesia (1.0 g/kg), a cuff-electrode was attached to the VN_left_ and vagotomy was performed peripheral to the stimulation site to isolate the afferent component of VNS by eliminating efferent fiber activation. Then, an eight-shank silicon probe (Buzsáki64) was targeted to PVT subregions. Only shanks that were properly identified by histological confirmation were included in analyses (Figure 2C, inset; Supplementary Figure S2A). Baseline spike rates before VNS (Pre period) in vagotomized mice were significantly lower in the pPVT compared with mice with no vagotomy (Supplementary Figure S2C; *n* = 117 and 69 cells from 5 mice each, *Z* = 2.50, *p* = 0.012; Mann–Whitney U test). After the Pre period, VNS (0.8 mA, 0.1 ms pulse width, 20 Hz, 10 pulses) was applied every 10 s for 10 min. VNS-excited and VNS-inhibited neurons were identified based on statistical comparisons of spike rates between the Pre and VNS periods (Figure 2D). Overall, these spike rate changes were transient during VNS and returned to baseline after VNS termination (Figure 2E). Neurons without significant changes in spike rates were classified as unchanged. In the aPVT, cPVT and pPVT, the proportions of excited and inhibited neurons were 17.9 and 2.6% (*n* = 39 cells), 23.1 and 9.2% (*n* = 65 cells), and 22.0 and 30.0% (*n* = 50 cells), respectively (Figure 2F). Statistically, while the proportion of VNS-excited neurons did not differ significantly among all PVT subregions (Figure 2F; aPVT vs. cPVT: *χ*^2^ = 0.38, *p* > 0.99; aPVT vs. pPVT: *χ*^2^ = 0.22, *p* > 0.99; cPVT vs. pPVT: *χ*^2^ = 0.02, *p* > 0.99, chi-square test followed by Bonferroni correction), the proportion of VNS-inhibited neurons in the pPVT was significantly higher than those in the aPVT and cPVT (aPVT vs. cPVT: *χ*^2^ = 1.73, *p* = 0.57; aPVT vs. pPVT: *χ*^2^ = 11.18, *p* = 0.0024; cPVT vs. pPVT: *χ*^2^ = 8.17, *p* = 0.013, chi-square test followed by Bonferroni correction). These results confirm that all PVT subregions contain VNS-excited neurons at similar proportions, whereas VNS-inhibited neurons are particularly enriched in the pPVT.

### PVT neurons show diverse spike responses to VN_left_-independent pathway activation

**Figure 3:**
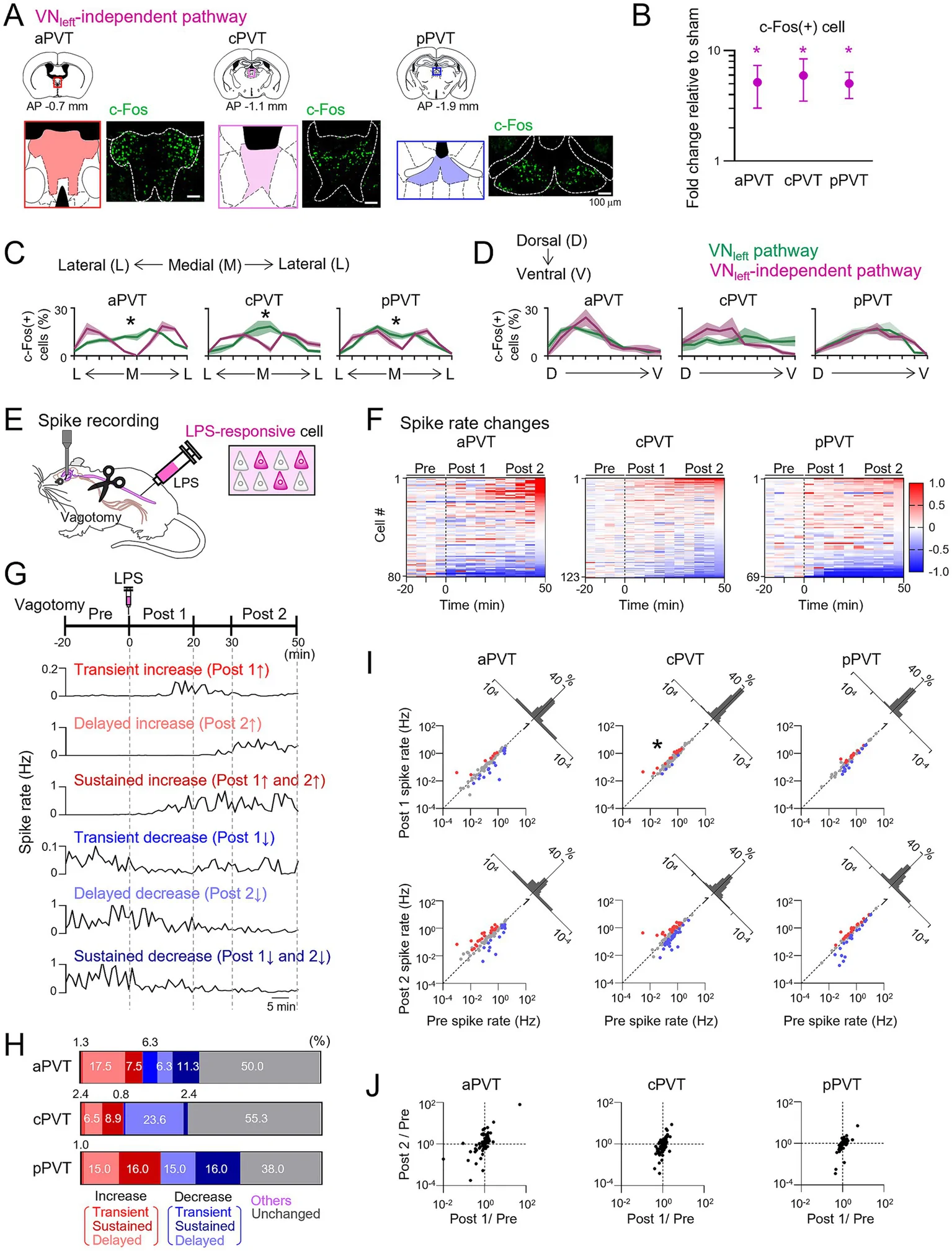
Spike responses of PVT neurons to VN_left_-independent pathway activation. **(A)** Pictures of c-Fos-expressing neurons after VN_left_-independent pathway activation (LPS injection after vagotomy). **(B)** Fold changes in c-Fos-positive neurons relative to sham controls (*n* = 9–11 sham and 10–12 LPS mice). Error bars represent SD. *p < 0.05, Mann–Whitney U test for aPVT and cPVT; Student’s *t*-test for pPVT. **(C)** Spatial distribution of c-Fos-positive neurons along the mediolateral axis in the aPVT, cPVT, and pPVT following VN_left_ (green) and VN_left_-independent (magenta) pathway activation. Each PVT subregion was divided into ten equally spaced bins along the mediolateral axis. Thick lines represent the mean percentage of c-Fos-positive neurons in each bin, and shaded areas indicate the SEM. * represents a significant difference in the spatial distribution between VN_left_ and VN_left_-independent pathway activation (*p* < 0.05). **(D)** Same as (C), but for the dorsoventral axis. **(E)** Schematic illustration of recording. **(F)** Heatmap of normalized spike rate changes. Neurons were aligned according to their rate changes at 50 min. PVT neuronal responses were recorded during VN_left_-independent pathway activation. **(G)** Representative neurons classified into different types based on LPS-induced spike rate changes. **(H)** Proportions of neuron types in each PVT subregion (*n* = 80, 123, and 69 cells). **(I)** Scatter plots showing spike rates of individual neurons before (pre, *x*-axis) and after LPS administration (top, post 1; bottom, post 2; *y*-axis). * represents a significant increase in Post 1 (*p* < 0.05). In each panel, the histogram beyond the diagonal represents the distribution of the ratios of spike rates between the two periods (Post/Pre). **(J)** Scatter plot of normalized spike rates: post1/pre (*x*-axis) vs. post2/pre (*y*-axis). Each dot represents an individual neuron.

We next examined the effects of VN_left_-independent pathway activation (LPS injection after vagotomy) on PVT neurons. c-Fos expression analysis confirmed that all three PVT subregions showed significant increases in c-Fos-expressing neurons (Figures 3A,B; aPVT: *Z* = 3.91, *p* = 9.3 × 10^−5^; cPVT: *Z* = 3.94, *p* = 8.0 × 10^−5^, Mann–Whitney U test; pPVT: *t*_17_ = 6.31, *p* = 7.9 × 10^−6^, Student’s *t*-test). To determine whether VN_left_ (Figure 2A) and VN_left_-independent (Figure 3A) pathway activation differed in the spatial distribution of activated PVT neurons, the percentage of c-Fos-positive neurons was analyzed along the mediolateral (Figure 3C) and dorsoventral (Figure 3D) axes in the aPVT, cPVT, and pPVT. Along the mediolateral axis, the spatial distribution of neurons activated by the two pathways differed significantly in the aPVT, cPVT, and pPVT (aPVT, *p* = 0.00050; cPVT, *p* = 0.017; pPVT, *p* = 0.034, two-way repeated-measures ANOVA, Greenhouse–Geisser corrected). In all the subregions, activation of VN_left_ pathway induced a higher density of c-Fos-positive neurons, distributed more medially, compared with activation of the VN_left_-independent pathway. In contrast, no significant difference in the spatial distribution was detected along the dorsoventral axis in any PVT subregion (aPVT, *p* = 0.38; cPVT, *p* = 0.16; pPVT, *p* = 0.62, two-way repeated-measures ANOVA, Greenhouse–Geisser corrected). These results suggest that VN_left_ and VN_left_-independent pathways activate distinct PVT neuronal populations along the medial-lateral axis.

Similar to VNS in Figure 2, we conducted multiunit recordings to measure spike rate changes following VN_left_-independent pathway activation in vagotomized mice (Figure 3E). First, we constructed heatmaps showing spike rate changes of individual neurons in response to LPS injection (Figure 3F). Because LPS administration triggers various inflammatory responses and temporal changes in humoral factors, several neuronal responses developed over tens of minutes and exhibited diverse temporal dynamics across individual neurons. We therefore divided the post-LPS period into two phases: Post 1 (0–20 min) and Post 2 (30–50 min) (Figure 3G). Each neuron was classified based on statistical comparisons of spike rates between the Post 1/Post 2 and Pre periods: (1) transient increase/decrease: significant changes in Post 1 only, (2) delayed increase/decrease: significant changes in Post 2 only, (3) sustained increase/decrease: significant changes in both Post 1 and Post 2 periods. Neurons with mixed response patterns (e.g., increase in Post 1 and decrease in Post 2) were classified as others. Across all subregions, transiently responsive neurons represented a minor population (<10%), and the majority exhibited either delayed responses (activation emerging in Post 2) or sustained responses (activation beginning in Post 1 and persisting through Post 2). These results indicate that PVT neurons do not simply sense transient LPS-induced conditions and return to baseline, but rather continuously encode persistent LPS-induced states. Overall, the proportion of responsive neurons in the pPVT was higher than in the aPVT and cPVT (Figure 3H; aPVT vs. cPVT: *χ*^2^ = 0.72, *p* > 0.99; aPVT vs. pPVT: *χ*^2^ = 5.00, *p* = 0.076; cPVT and pPVT: *χ*^2^ = 10.39, *p* = 0.0038, chi-square test followed by Bonferroni correction), indicating that the pPVT is most responsive to VN_left_-independent pathway activation across PVT subregions.

To assess overall changes in spike rates of neuronal populations, we compared spike rates between the Pre and Post periods (Figure 3I). In the cPVT, but not in the aPVT and pPVT, spike rates during the Post 1 period were significantly increased, compared with the Pre period (aPVT: *n* = 80 cells*, t_79_* = 1.79, *p* = 0.076; cPVT: *n* = 123 cells*, t_122_* = 2.89, *p* = 0.0045; pPVT: *n* = 69 cells*, t_68_* = 0.96, *p* = 0.34, paired *t*-test). However, during the Post 2 period, no significant changes in spike rates were observed from the Pre period in any subregion (aPVT: *t_79_* = 1.25, *p* = 0.21; cPVT: *t_122_* = 0.73, *p* = 0.46; pPVT: *t_68_* = 0.33, *p* = 0.73, paired *t*-test). These results demonstrate that while individual PVT neurons show either increased or decreased spike rates following LPS administration (Figure 3H), these opposing changes are balanced at the population level, with no net directional bias in overall activity. The same experiment and analysis were performed in mice with the intact VN_left_ (Supplementary Figure S1B). During the Post 1 period, no significant changes in spike rates were observed in any PVT subregion (Supplementary Figure S1C; aPVT: *n* = 76 cells*, t_75_* = 0.39, *p* = 0.69; cPVT: *n* = 111 cells*, t_110_* = 0.0016, *p* = 0.99; pPVT: *n* = 117 cells*, t_116_* = 0.77, *p* = 0.46, paired *t*-test). During the Post 2 period, spike rates were significantly decreased in the aPVT and pPVT (aPVT: *t_75_* = 2.09, *p* = 0.040; cPVT: *t_110_* = 1.67, *p* = 0.098; pPVT: *t_116_* = 2.83, *p* = 0.0054, paired *t*-test). These results suggest that the VN_left_ pathway suppresses neuronal activity in these PVT subregions during the sustained phase following LPS administration.

To further assess the strength of LPS-induced changes in spike activity through the VN_left_-independent pathway, we compared the distributions of spike rate change ratios relative to the Pre period (upper-right inset of Figure 3I). During the Post 1 period, absolute rate changes in the aPVT were significantly larger than those in the cPVT (aPVT vs. cPVT: *p* = 0.014; aPVT vs. pPVT: *p* = 0.95; cPVT vs. pPVT*, p* = 0.082, Dunn’s multiple-comparison test following a Kruskal–Wallis test). During the Post 2 period, no significant differences in absolute rate changes were observed among PVT subregions (aPVT vs. cPVT: *p* = 0.12; aPVT vs. pPVT: *p* = 0.61; cPVT vs. pPVT: *p* = 0.85, Dunn’s multiple-comparison test following a Kruskal–Wallis test). These results suggest that the magnitude of population-level activity changes does not differ across PVT subregions in the tens of minutes following LPS administration.

We next examined the relationship of spike rate change ratios between the Post 1 and Post 2 periods (Post 1/Pre vs. Post 2/Pre) during VN_left_-independent activation. In all PVT subregions, these two ratios were significantly correlated (Figure 3J; aPVT: Spearman’s *ρ* = 0.60, *p* = 3.5 × 10^−9^; cPVT: *ρ* = 0.61, *p* = 5.3 × 10^−14^; pPVT: *ρ* = 0.79, *p* = 1.4 × 10^−15^). The same results were observed from intact VN_left_ mice (Supplementary Figure S1D; aPVT: *ρ* = 0.75, *p* = 3.0 × 10^−15^; cPVT: *ρ* = 0.75, *p* = 4.1 × 10^−21^; pPVT: *ρ* = 0.57, *p* = 3.1 × 10^−11^). These results demonstrate temporal consistency in the direction of spike rate modulation following LPS administration, as neurons that increased their spike rates during Post 1 tended to continue increasing during Post 2, whereas neurons that decreased their spike rates tended to remain suppressed.

PVT neurons are predominantly glutamatergic, but heterogeneous in terms of gene expression and physiological properties, which likely contribute to their functional responses. We therefore assessed whether these basic electrophysiological characteristics of individual neurons were associated with their response patterns to VN_left_-independent pathway activation. The relationships between each electrophysiological property (spike half-width, peak-to-trough ratio, baseline firing rate, and burstiness index) and the spike rate change ratio (Post 2/Pre) were examined (Supplementary Figure S3). For spike half-width and burstiness index, neurons showing decreased spike rates exhibited significantly lower values than neurons showing increased spike rates in the cPVT and pPVT (Supplementary Figures S3A,D). For peak-to-trough ratio, neurons showing decreased spike rates exhibited significantly lower ratios than neurons showing increased spike rates in the cPVT (Supplementary Figure S3B). For baseline spike rates before VN_left_-independent pathway activation, no significant differences were found among neurons showing increased, decreased, and unchanged spike rates (Supplementary Figure S3C). Taken together, neurons with decreased spike rates in response to VN_left_-independent pathway activation exhibit distinct electrophysiological characteristics, including smaller spike half-width, lower peak-to-trough ratio, and lower burstiness index in the cPVT and pPVT, suggesting that PVT neurons with distinct spike properties exhibit responses in different directions of spike rate change to interoceptive mechanisms.

### Distinct PVT neuronal ensembles are activated by the VN_left_ and VN_left_-independent pathways

**Figure 4:**
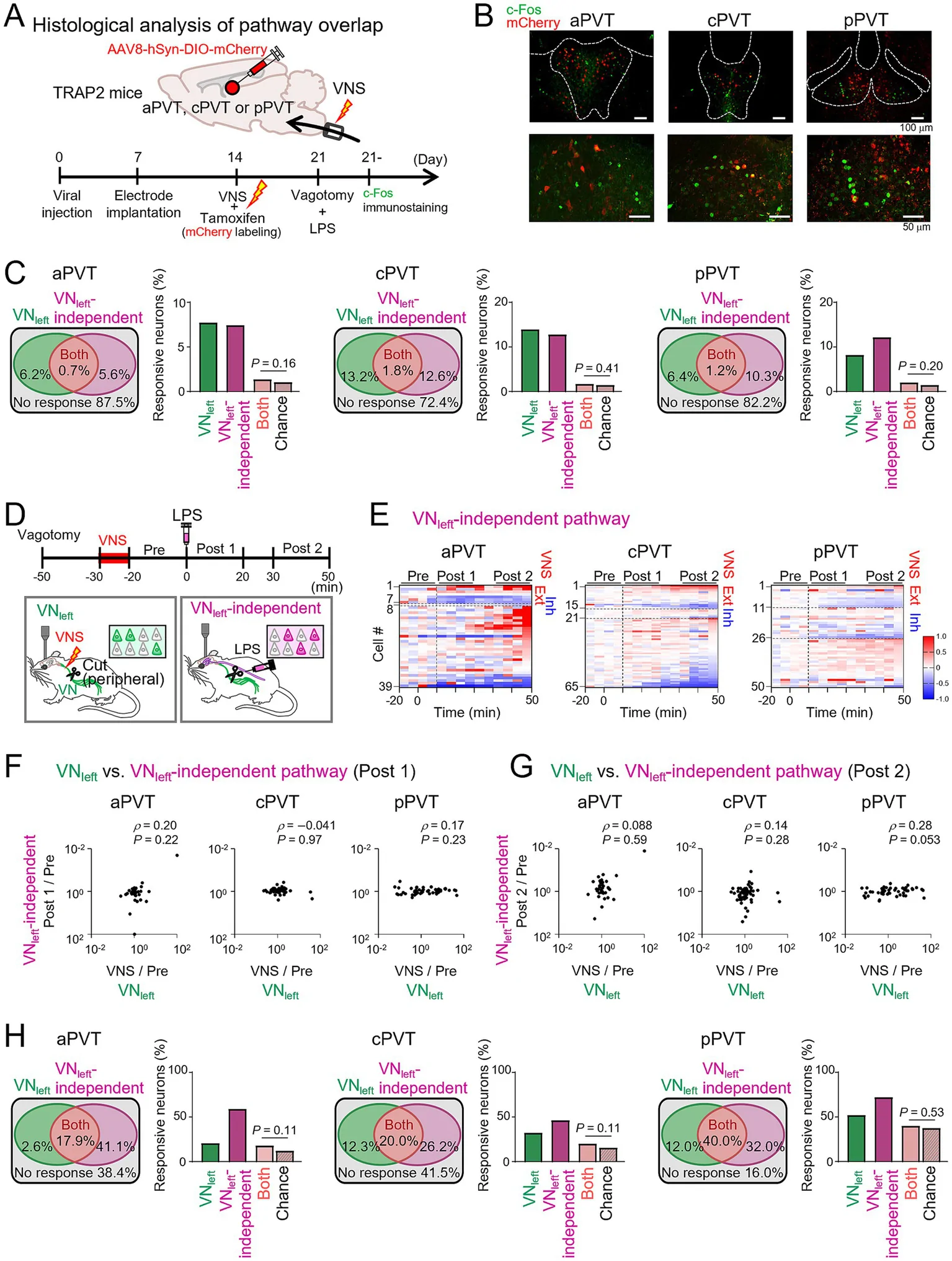
Relationship of PVT neuronal spike responses between VN_left_ and VN_left_-independent pathway activation. **(A)** Using the TRAP2 system, VNS-responsive neurons in the PVT (aPVT, cPVT or pPVT) were selectively labeled with mCherry. Following vagotomy and LPS injection, VN_left_-independent pathway-responsive neurons were identified by c-Fos immunostaining. **(B)** Representative images showing mCherry-labeled VNS-responsive neurons (red) and c-Fos-positive neurons activated by the VN_left_-independent pathway (green) in the aPVT, cPVT, and pPVT. **(C)** overlap analysis of neurons activated by the VN_left_ and VN_left_-independent pathways in the aPVT, cPVT, and pPVT. For each PVT subregion, left panels show Venn diagrams illustrating the overlap between neurons activated by the two pathways, and right panels show the proportions of neurons activated by the VN_left_ pathway, VN_left_-independent pathway, both pathways, and the expected overlap by chance (*n* = 1,503, 840, and 1,245 total cells from 2–3 mice for the aPVT, cPVT, and pPVT, respectively). **(D)** In a mouse, after performing vagotomy peripheral to the stimulation site and placing electrodes in the PVT, 10-min VNS was first applied as vagal activation, and LPS was then injected as VN_left_-independent pathway activation. **(E)** Heatmap of normalized spike rate changes. Neurons were aligned according to their cell types in response to VNS (VNS-excited, VNS-inhibited, and or unchanged neurons; *n* = 39, 65, and 50 cells from 6, 5, and 3 mice, respectively). **(F)** Relationship between spike rate changes in response to VN_left_ pathway activation (x-axis) and those to VN_left_-independent pathway activation during the Post 1 period. Each dot represents an individual neuron. **(G)** Similar to **(F)**, but for the Post 2 period. **(H)** Similar to **(C)**, overlap analysis of electrophysiologically identified neurons responsive to the VN_left_ and VN_left_-independent pathways in the aPVT, cPVT, and pPVT.

We further examined whether VN_left_ and VN_left_-independent pathway activation engages overlapping or distinct neuronal populations using the TRAP2 system, in which activated neurons are genetically labeled in a manner dependent on c-Fos promoter activity. In Fos^TRAP^ mice, Cre-dependent AAV8-hSyn-DIO-mCherry was injected into either the aPVT, cPVT or pPVT. Two weeks later, mice received tamoxifen followed by VNS to label VNS-activated neurons. Subsequently, mice underwent vagotomy, followed by LPS injection. c-Fos immunostaining was then performed to identify neurons activated by the VN_left_-independent pathway (Figures 4A,B). VNS activated 6.9, 15.0, and 7.6% of cells in the aPVT, cPVT, and pPVT, respectively, whereas LPS activated 6.3, 14.4, and 11.5% of cells in the corresponding subregions (Figure 4C). Based on these activation rates, the expected overlap by chance was 0.4, 2.2, and 0.9% in the aPVT, cPVT, and pPVT, respectively. However, the observed overlap was 0.7, 1.8, and 1.2%, which did not differ significantly from the chance labels (aPVT, *p* = 0.16; cPVT, *p* = 0.41; pPVT, *p* = 0.20, Fisher’s exact test). These results demonstrate that the proportion of neurons responsive to both pathways did not exceed the level predicted from each proportion of neurons activated by each single pathway.

To further examine the electrophysiological properties of these neuronal populations, we first applied VNS for 10 min in mice with vagotomy peripheral to the stimulation site (as in Figure 2) to identify VN_left_-responsive PVT neurons (Figure 4D). Subsequently, we activated the VN_left_-independent pathway via LPS administration (as in Figure 3) and examined neuronal responses in the identical neurons. Each cell type identified by VN_left_ pathway activation (VNS-excited or VNS-inhibited) showed diverse temporal dynamics during the Post 1 and Post 2 periods of VN_left_-independent pathway activation (Figure 4E). To quantify these responses, we plotted VN_left_ pathway-induced spike change ratios (VNS/Pre) against VN_left_-independent pathway-induced ratios (Post/Pre) for each neuron (Figures 4F,G). No significant correlation was observed between Post 1 ratios and VNS-induced ratios in any PVT subregion (Figure 4F; aPVT: Spearman’s *ρ* = 0.20, *p* = 0.22; cPVT: *ρ* = − 0.041, *p* = 0.97; pPVT: *ρ* = 0.17, *p* = 0.23). Similarly, Post 2 ratios showed no significant correlation with VNS-induced ratios in any PVT subregion (Figure 4G; aPVT: *ρ* = 0.088, *p* = 0.59; cPVT: *ρ* = 0.14, *p* = 0.28; pPVT: *ρ* = 0.28, *p* = 0.053). To further characterize the overlap between neuronal populations responsive to the VN_left_ and VN_left_-independent pathways, the proportion of neurons responsive to both pathways was quantified and compared with the overlap expected by chance (Figure 4H). For this analysis, neurons showing either increased or decreased spike activity in response to either pathway were classified as responsive neurons. For VN_left_-independent pathway activation, neurons showing significant changes in spike activity during either the Post 1 or Post 2 period were classified as responsive neurons. VNS activated 20.5, 32.3, and 52.0% of cells in the aPVT, cPVT, and pPVT, respectively, whereas LPS activated 59.0, 46.2, and 72.0% of cells in the corresponding subregions. Based on these activation rates, the expected overlap by chance was 12.1, 14.9, and 37.4% in the aPVT, cPVT, and pPVT, respectively. However, the observed overlap was 17.9, 20.0, and 40.0%, which did not differ significantly from the chance labels (Fisher’s exact test: aPVT, *p* = 0.11; cPVT, *p* = 0.11; pPVT, *p* = 0.53). These results further suggest that VN_left_ and VN_left_-independent pathways predominantly recruit distinct neuronal populations.

### VN_left_-responsive PVT neurons project to limbic emotion-related regions

**Figure 5:**
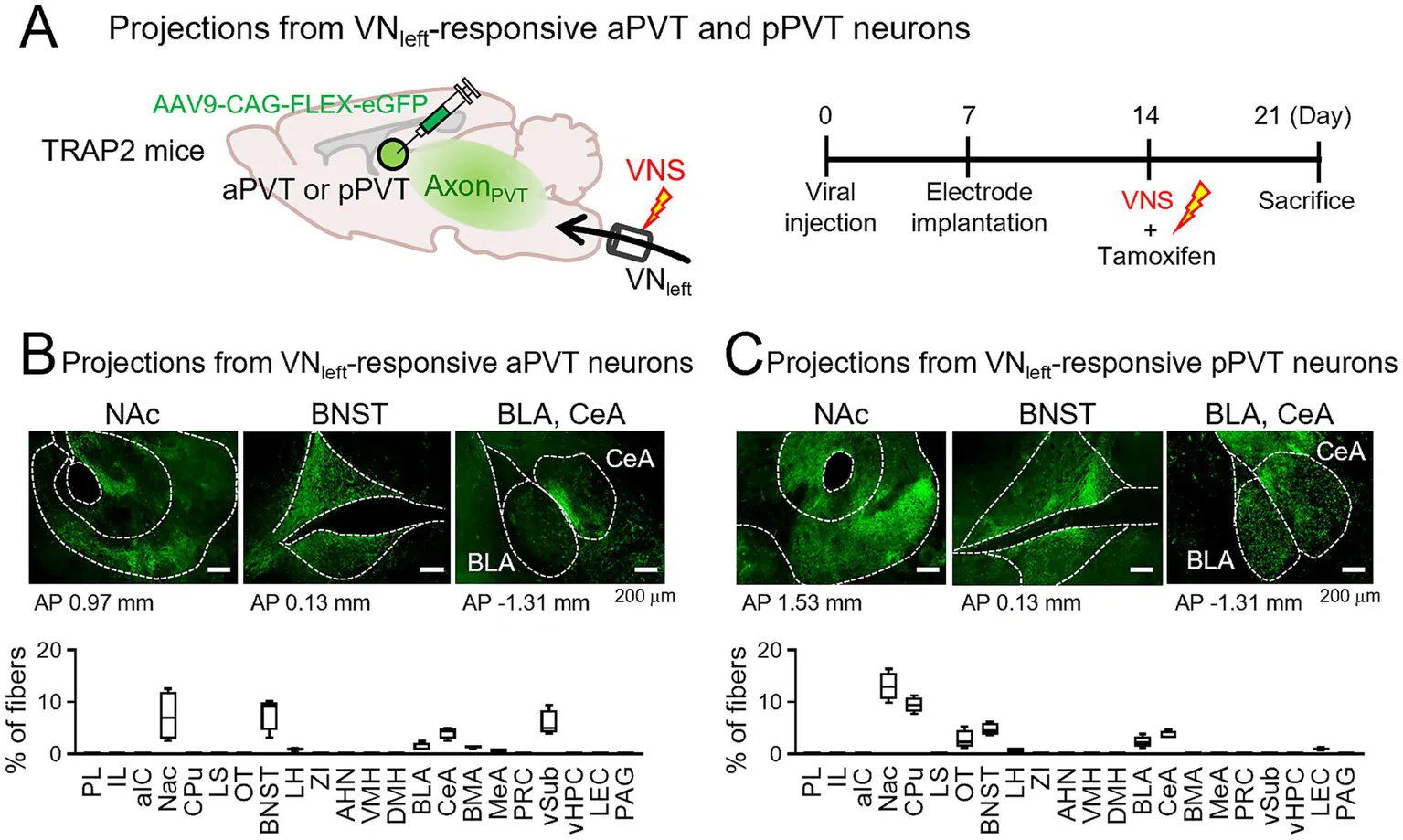
Projection patterns of VNS-responsive PVT neurons. **(A)** Using the TRAP2 system, VNS-responsive neurons in the PVT (aPVT or pPVT) were selectively labeled with GFP, and their axonal projections were subsequently traced. **(B)** (Top) Representative images showing GFP-expressing neurons in the aPVT and their axonal projections in downstream regions. (Bottom) Projection density of axonal fibers across brain regions for VNS-responsive aPVT neurons. Box plots show the center line as the median, box limits as the 25th and 75th percentiles, and whiskers as the minimum and maximum values excluding outliers (*n* = 4 mice). **(C)** Same as B, but for the pPVT (*n* = 4 mice).

We next examined the downstream projection targets of VN_left_-responsive PVT neurons using the TRAP2 system. In Fos^TRAP^ mice, Cre-dependent AAV9-CAG-FLEX-eGFP was injected into either the aPVT or pPVT (Figure 5A). After 2 weeks, mice received tamoxifen followed by VNS to label activated neurons. VN_left_-responsive aPVT neurons sent GFP-positive axonal projections to the nucleus accumbens (NAc), bed nucleus of the stria terminalis (BNST), central amygdala (CeA), and ventral subiculum (vSub), with weaker projections to the lateral hypothalamus (LH), basolateral amygdala (BLA), basomedial amygdala (BMA), and medial amygdala (MeA) (Figure 5B). In contrast, VN_left_-responsive pPVT neurons projected to the NAc, caudate putamen (CPu), olfactory tubercle (OT), BNST, BLA, and CeA, with weaker projections to the LH and lateral entorhinal cortex (LEC) (Figure 5C). No projections were observed in the prelimbic cortex (PL), infralimbic cortex (IL), anterior insular cortex (aIC), lateral septum (LS), zona incerta (ZI), anteromedial hypothalamus (AHN), ventromedial hypothalamus (VMH), dorsomedial hypothalamus (DMH), perirhinal cortex (PRC), PAG, and ventral hippocampus (vHPC) in either aPVT or pPVT neurons. These results indicate that VN_left_-responsive PVT neurons project to limbic regions related to emotional processing.

### Inhibition of VN_left_-responsive PVT neurons induces anxiety-like behavior

**Figure 6:**
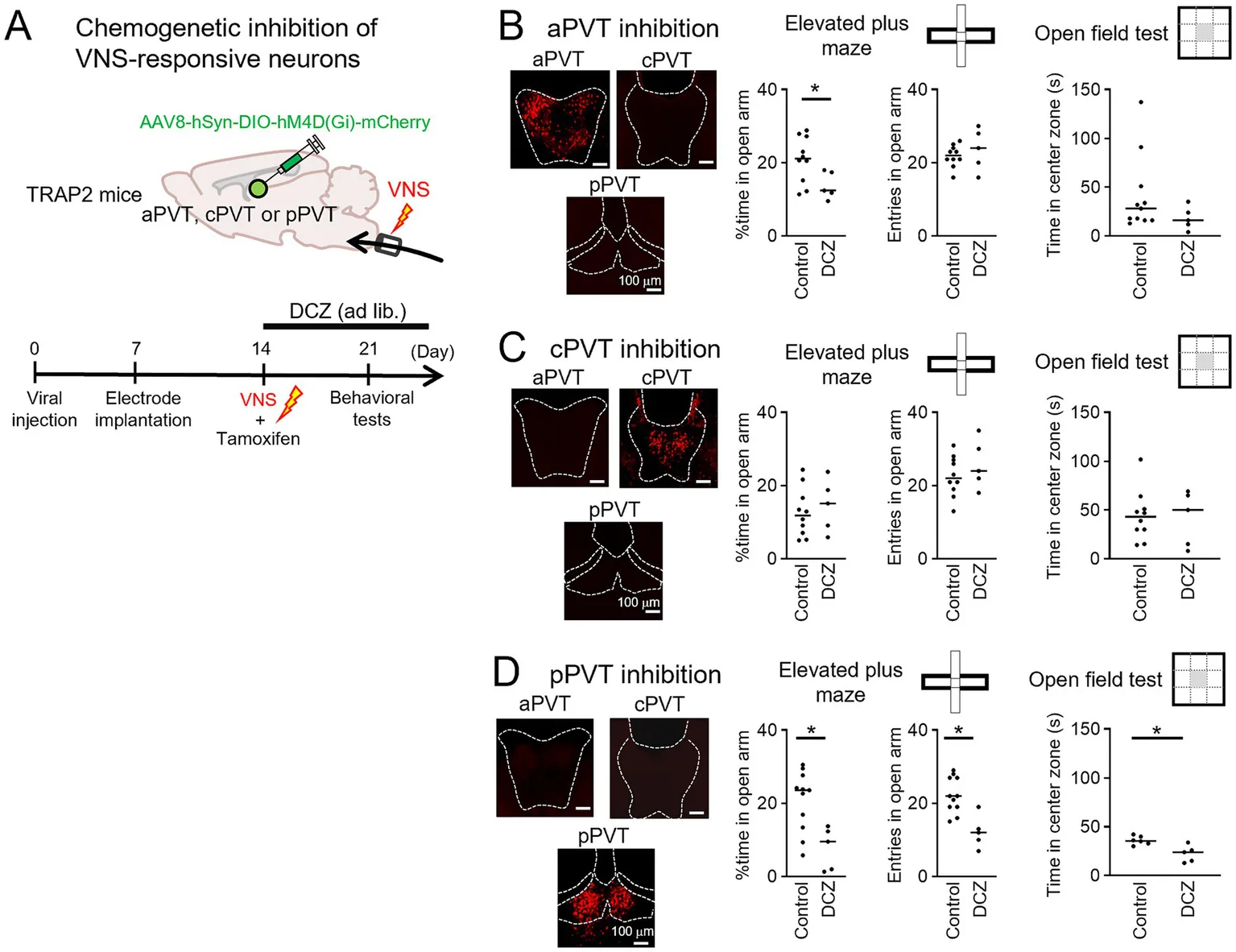
Chemogenetic inhibition of VNS-responsive PVT neurons induces anxiety-like behavior. **(A)** Using the TRAP2 system, VNS-activated neurons in the PVT (aPVT, cPVT, or pPVT) were labeled with hM4Di and subsequently chemogenetically inhibited during behavioral testing. Control mice underwent identical procedures but did not receive DCZ. **(B)** Behavioral results from mice with chemogenetic inhibition of VNS-responsive aPVT neurons. (Left) Representative images of mCherry-expressing neurons in the aPVT, cPVT, and pPVT. (Middle) Elevated plus maze performance, showing the percentage of time spent in the open arms (left) and the number of open arm entries (right) during the total recording period (*n* = 10 and 5 mice for control and DCZ groups, respectively). *p < 0.05, Student’s *t*-test. (Right) Open field test performance, showing the time spent in the center zone during the total recording period (*n* = 11 and 5 mice). **(C)** Same as B, but for cPVT neurons (Elevated plus maze: *n* = 10 and 5 mice; Open field test: *n* = 10 and 5 mice). **(D)** Same as B, but for pPVT neurons (Elevated plus maze: *n* = 11 and 5 mice; Open field test: *n* = 6 and 5 mice). *p < 0.05, Student’s *t*-test.

To test the contribution of VN_left_-responsive PVT neurons to behavior, we performed chemogenetic inhibition of VNS-induced neurons using the TRAP2 system. In Fos^TRAP^ mice, Cre-dependent AAV8-hSyn-DIO-hM4Di-mCherry was injected into the aPVT, cPVT, or pPVT (Figure 6A). After 2 weeks, mice received a tamoxifen injection followed by VNS to label VN_left_-responsive neurons with hM4Di. Labeled neurons were specifically restricted to the injected subregion (aPVT, cPVT, or pPVT) (Figures 6B–D). Subsequently, mice were given free access to DCZ-containing water for 1 week to inhibit the labeled neuronal ensembles. Anxiety-like behavior was then evaluated using an elevated plus maze test and an open field test. Compared with a control mouse group (mice that underwent identical procedures but received water instead of DCZ), the aPVT inhibition mouse group with DCZ showed a significant decrease in time spent in the open arms (Figure 6B; *n* = 10 and 5 mice for control and DCZ groups; *t_13_* = 2.99, *p* = 0.039, Student’s *t*-test), but no change in the number of open arm entries in the elevated plus maze (*t_13_* = 0.77, *p* = 0.45, Student’s *t*-test). In the open field test, no significant differences in time spent in the center zone were observed between the two mouse groups (*n* = 11 and 5 mice for control and DCZ groups, *Z* = −1.36*, p* = 0.17, Mann–Whitney U test). Compared with a control group, the cPVT inhibition mouse group showed no change in the time spent in the open arms (Figure 6C; *n* = 10 and 5 mice for control and DCZ groups; *t_13_* = 0.51, *p* = 0.62, Student’s *t*-test) or the number of open arm entries in the elevated plus maze (*n* = 10 and 5 mice for control and DCZ groups, *t_13_* = 0.99, *p* = 0.34, Student’s *t*-test). In the open field test, no significant differences in time spent in the center zone were observed between the two mouse groups (*n* = 10 and 5 mice for control and DCZ groups, *t_13_* = 0.18, *p* = 0.86, Student’s *t*-test). Compared with a control group, the pPVT inhibition mouse group showed significant decreases in time spent in the open arms (Figure 6D; *n* = 11 and 5 mice for control and DCZ groups; *t_14_* = 3.13, *p* = 0.0073, Student’s *t*-test) and the number of open arm entries in the elevated plus maze (*t_14_* = 3.93, *p* = 0.0015, Student’s *t*-test). In the open field test, the pPVT inhibition group showed a significant decrease in time spent in the center zone (*n* = 6 and 5 mice for control and DCZ groups, *t_9_* = 3.41*, p* = 0.0078, Student’s *t*-test). These results suggest that VN_left_-responsive PVT neurons in the aPVT and pPVT are involved in the regulation of anxiety-like behavior. In addition, the PVT has been implicated in appetite regulation (Meffre et al., 2019; Shima et al., 2023). We therefore compared food consumption between the control and the PVT inhibition groups (Supplementary Figure S4A). The pPVT inhibition group showed a significant decrease in food consumption, whereas the aPVT and cPVT inhibition groups did not show such a significant change (Supplementary Figure S4B). These results suggest that VN_left_-responsive pPVT neurons promote food intake.

## Discussion

In this study, we found that PVT neurons showed increased c-Fos expression after activation of both VN_left_ and VN_left_-independent pathways. Multiunit spike recordings revealed that VN_left_ activation induces transient increases and decreases in neuronal spike rates across all PVT subregions. VN_left_-independent pathway activation by LPS injection following vagotomy also induced bidirectional changes in spike rates of PVT neurons, with temporal dynamics ranging from transient to delayed and sustained responses. The patterns of neuronal spike rate changes induced by VN_left_ and VN_left_-independent pathway activation were not correlated across any PVT subregion. Using the TRAP2 system, we demonstrated that VN_left_-responsive PVT neurons project to limbic structures, including the NAc, BNST, and CeA. Consistent with these projection patterns, inhibition of these neuronal ensembles in the aPVT and pPVT induced anxiety-like behavior, suggesting that VN_left_-responsive PVT neurons play a key role in controlling anxiety.

### PVT neurons respond to VN_left_ pathways in addition to humoral pathways

Our results demonstrate that PVT neurons are critically involved in VN_left_ signaling. Combined axonal tracing suggested that this pathway is mediated by direct projections from the cNTS, a primary relay of VN_left_ afferents, as well as indirect projections via brainstem nuclei, including the LC, PBN, and PAG. Previous studies have shown that the VN-PBN-PVT pathway contributes to the transmission of visceral nociceptive signals, suggesting that this pathway is a route by which aversive interoceptive information reaches the PVT (Zhang et al., 2024). Given its location adjacent to the third ventricle, the PVT is well positioned to sense humoral signals circulating in the cerebrospinal fluid, and may thus integrate two distinct interoceptive streams, vagal afferents and circulating humoral signals. Consistent with this notion, we demonstrated that the PVT was uniquely activated by both the VN_left_ pathway and the VN_left_-independent pathway, including the humoral pathway, which were not observed in any other brain region examined. Taken together, our findings suggest that the PVT serves as an integrative hub to collect diverse interoceptive signals and process their information through varied neuronal spike modulation patterns with high temporal precision.

### Distinct spike patterns of PVT neurons in response to the VN_left_ and VN_left_-independent pathways

Our multiunit spike recordings demonstrated that the PVT, irrespective of subregion, contains diverse neurons exhibiting not only increased but also decreased spike rates in response to both VN_left_ pathway activation and VN_left_-independent pathway activation. Such bidirectional spike response patterns are considered to arise primarily from upstream regions providing input to the PVT. According to our results (Figure 1B) and previous studies [e.g., (Biro et al., 2025)], candidate sources of VN-related information include direct input from the NTS, as well as indirect input relayed through intermediary regions such as the LC, PBN, and PAG. Projections from these regions to the PVT are likely to comprise a mixture of excitatory and inhibitory inputs, which are considered to generate the bidirectional spike responses observed in specific PVT neurons. Consistent with this idea, a recent study identified long-range GABAergic projections from the NTS to the PVT (Shi et al., 2021), providing a potential mechanism for the inhibitory responses observed in our recordings. Inhibitory afferents from other regions, such as the zona incerta and PAG (Biro et al., 2025), may also contribute to the suppression of PVT neuronal activity. Although the PVT contains a small population of inhibitory neurons, these neurons might also contribute to inhibitory responses through a feedforward mechanism.

Outputs from these diverse PVT neurons to downstream regions are also important. Using the TRAP2 system, we identified candidate downstream regions including the NAc, BNST, and CeA (Figure 5). Given that the majority of PVT neurons are excitatory, and that cells labeled using the TRAP method were putatively activated neurons, these downstream regions receive predominantly excitatory influence from the PVT. At the same time, whether inhibited PVT neurons project to the same downstream regions remains unclear. If both types of PVT neurons project to the same downstream regions, a diversity of increased and decreased inputs needs to be considered at the level of individual cells and fibers. Overall, the coexistence of stimulus-induced neuronal excitation and inhibition is considered to enhance the signal-to-noise ratio of information processing in response to diverse interoceptive signals.

In addition, our c-Fos analysis revealed distinct mediolateral spatial distributions of neurons responsive to the VN_left_ and VN_left_-independent pathways. Furthermore, TRAP2-based overlap analysis demonstrated that the proportion of neurons activated by both pathways did not differ significantly from that predicted by their individual activation rates. Consistent with these findings, when the VN_left_ and VN_left_-independent pathways were sequentially activated, no significant correlations or overlaps were observed between the direction or magnitude of spike rate changes evoked by the two pathway activations. Together, these results suggest that distinct neuronal populations encode interoceptive signals arising from these two pathways, further enhancing computational capacity in the PVT-related circuit for their segregated processing.

The time scales of spike responses to activation of the two pathways were diverse. The VNS-induced PVT neuronal responses occurred rapidly (within a minute), consistent with a nerve-mediated mechanism. Given that the VN innervates a wide range of visceral organs, including the heart and gut, these findings suggest that vagal afferents play an important role in rapidly conveying information of visceral organ states to PVT neurons. On the other hand, VN_left_-independent pathway activation induced diverse changes in PVT neuronal spike rates over several tens of minutes, exhibiting transient, delayed, and sustained patterns. The diversity of temporal response patterns cannot be simply accounted for by the delayed effects of intraperitoneally administered LPS. During inflammatory states induced by LPS administration, peripheral physiological conditions are considered to change across multiple timescales. The VN_left_-independent pathway, including humoral pathways transmitting circulating factors such as cytokines, likely conveys these signals to the PVT across diverse timescales, and our results suggest that PVT neurons represent this multiplexed input through their diverse temporal spike patterns. Taken together, our results suggest that PVT neurons are capable of encoding distinct spatiotemporal interoceptive signals conveyed through vagal and vagal-independent pathways.

### The contribution of VN_left_-responsive PVT neurons to emotion-related circuits and behavior

The PVT predominantly consists of glutamatergic neurons with sparse recurrent connectivity (Gao et al., 2020; Ye et al., 2022), while including very few GABAergic interneurons (Gao et al., 2023; Shima et al., 2023). These anatomical features suggest that PVT neurons activated by interoceptive signals primarily provide excitatory inputs to downstream brain areas. While PVT neurons have been reported to project broadly to limbic regions and higher-order cortical areas including the prefrontal cortex, insular cortex, and ventral subiculum (Moga et al., 1995; Li and Kirouac, 2008; Li et al., 2021; Shima et al., 2023), our anterograde tracing using the TRAP2 system revealed that VN_left_-responsive PVT neurons preferentially targeted emotion-related limbic regions, particularly the NAc, BNST, and CeA, with comparatively sparse projections to such cortical regions. These findings suggest that vagal signals conveyed to the PVT are mainly routed toward emotion-related limbic functions rather than higher-order cortical processing.

Consistent with this notion, we demonstrated that inhibition of VN_left_-responsive PVT neurons enhanced anxiety-like behavior. Accumulating evidence indicates that vagotomy and VNS affect neuronal activity across a wide range of brain regions, resulting in diverse effects on emotional behavior (Breit et al., 2018; Johnson and Wilson, 2018; Noble et al., 2019; Wang et al., 2021; Okonogi et al., 2024). Our findings, derived from a more selective dissection of PVT neuron contributions, indicate that vagally driven signals processed within the PVT are associated with anxiety-related emotional states.

### Subregional differences between the aPVT and pPVT

We demonstrated that the pPVT contained larger proportions of neurons responding to both VN_left_ and VN_left_-independent pathway activation compared to the aPVT. This subregional difference may be partly explained by recent insights demonstrating that gene expression profiles of PVT neurons vary gradually along the anterior–posterior axis (Gao et al., 2020; Shima et al., 2023). However, the physiological differences found in our studies were modest, as neurons exhibiting similar spike responses were observed in both PVT subregions, suggesting that VN_left_-responsive neuronal ensembles mostly share a common functional role across PVT subregions. Consistent with these cellular-level characteristics, our behavioral analyses demonstrated that inhibition of VN_left_-responsive neurons in both the aPVT and pPVT produced anxiety-like behavior. While previous behavioral studies have demonstrated subregional differences, with the pPVT more strongly implicated in emotional regulation and the aPVT associated with arousal level (Barson et al., 2020; Iglesias and Flagel, 2021; Li et al., 2025), our findings suggest that, when these analyses are restricted to VN_left_-responsive neurons, such functional distinctions are less apparent.

## Conclusion

In summary, we identified the PVT as a convergent node that responds to both VN_left_ and VN_left_-independent interoceptive pathways. Multiunit recordings revealed that these two pathways activate distinct neuronal populations within the PVT. Furthermore, VN_left_-activated PVT neurons preferentially projected to limbic regions, and their inhibition produced anxiety-related behavioral effects. Together, these findings suggest that PVT neuronal populations integrate and relay interoceptive signals across multiple pathways and timescales to emotion-related circuits, thereby playing a pivotal role in selectively and precisely linking specific bodily states to distinct emotional behaviors.

## Data Availability

The original contributions presented in the study are included in the article/Supplementary material, further inquiries can be directed to the corresponding author.
