# Supplementary material for: Paraventricular thalamic neurons respond to vagus nerve activation and modulate anxious behavior
Source: Front Neural Circuits. 2026 Aug 24;20:1865790. doi: 10.3389/fncir.2026.1865790 (PMC13547499; doi:10.3389/fncir.2026.1865790)
Supplement: Supplementary file 1 [file Data_Sheet_1.PDF]

Supplementary Figures

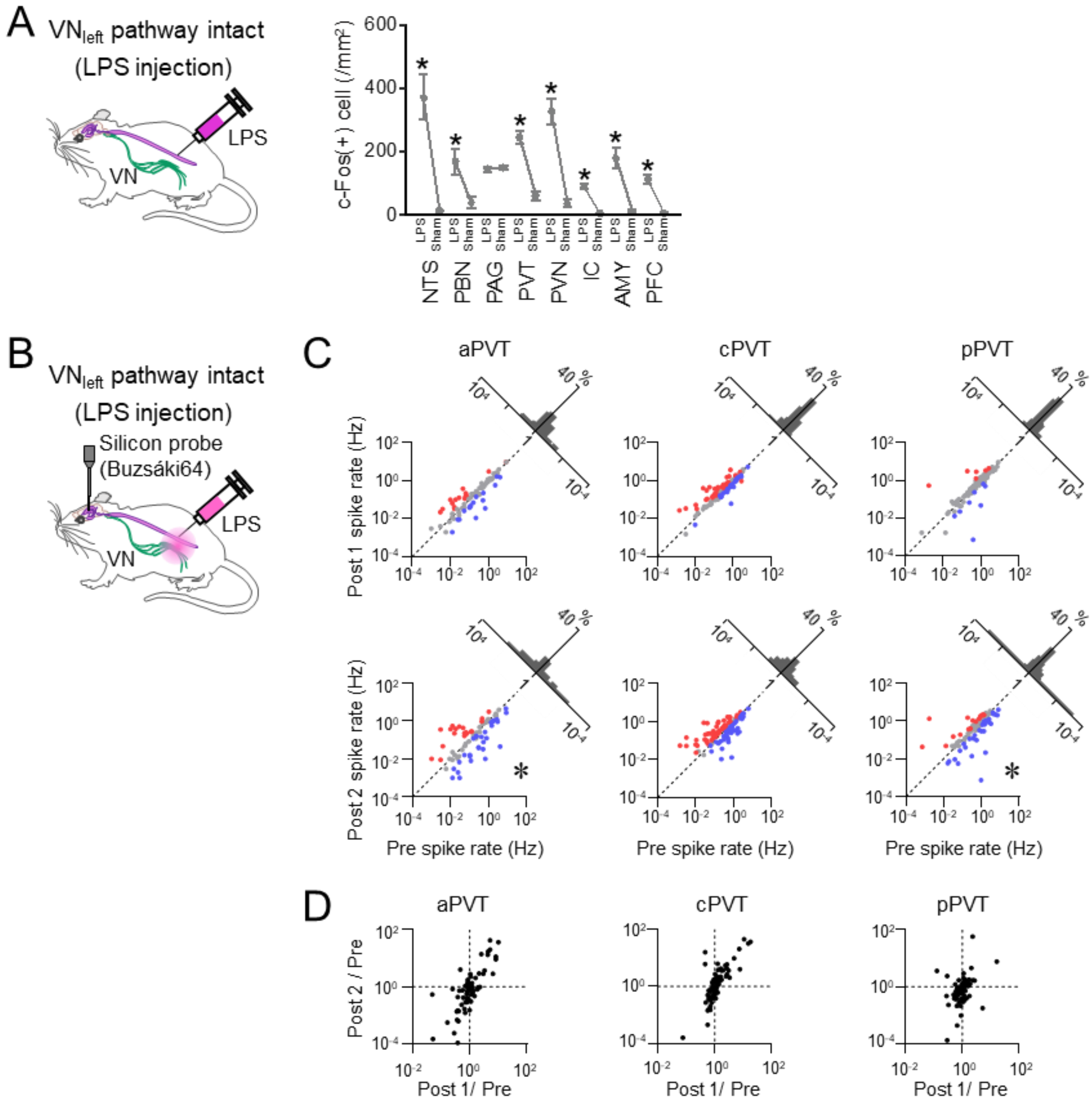

Supplementary Figure 1 Results from LPS administration in mice with both pathways intact. (A) LPS was injected without vagotomy (both pathways intact). The densities of c-Fos-positive neurons were compared with sham controls (*n* = 5–7 sham and 5–9 LPS mice). Error bars represent SEM. NTS: $t_{13} = 4.08$, $P = 0.0013$; PBN: $t_8 = 3.01$, $P = 0.017$; PAG: $t_{13} = 0.54$, $P = 0.60$; PVT: $t_{13} = 7.15$, $P = 7.4 \times 10^{-6}$; PVN: $t_{12} = 6.30$, $P = 3.9 \times 10^{-5}$; IC: $t_{10} = 12.35$, $P = 2.2 \times 10^{-7}$; AMY: $t_{12} = 4.53$, $P = 6.8 \times 10^{-4}$, PFC: $t_{10} = 7.62$, $P = 1.8 \times 10^{-5}$, Student's *t*-test. **(B)** Multiunit recording was performed during LPS injection in mice with the intact VN$_{left}$. **(C)** Scatter plots showing spike rates of individual neurons before (Pre, x-axis) and after LPS injection (post1 or post2, y-axis) (*n* = 76, 110, and 117 cells from 11, 11,

and 5 mice, respectively). * represents significant decreases in Post 2 ($P < 0.05$). In each panel, the histogram beyond the diagonal represents the distribution of the ratios of spike rates between the two periods (Post/Pre). **(D)** Scatter plots showing normalized spike rate ratios (post1/pre on x-axis, post2/pre on y-axis). Each dot represents an individual neuron.

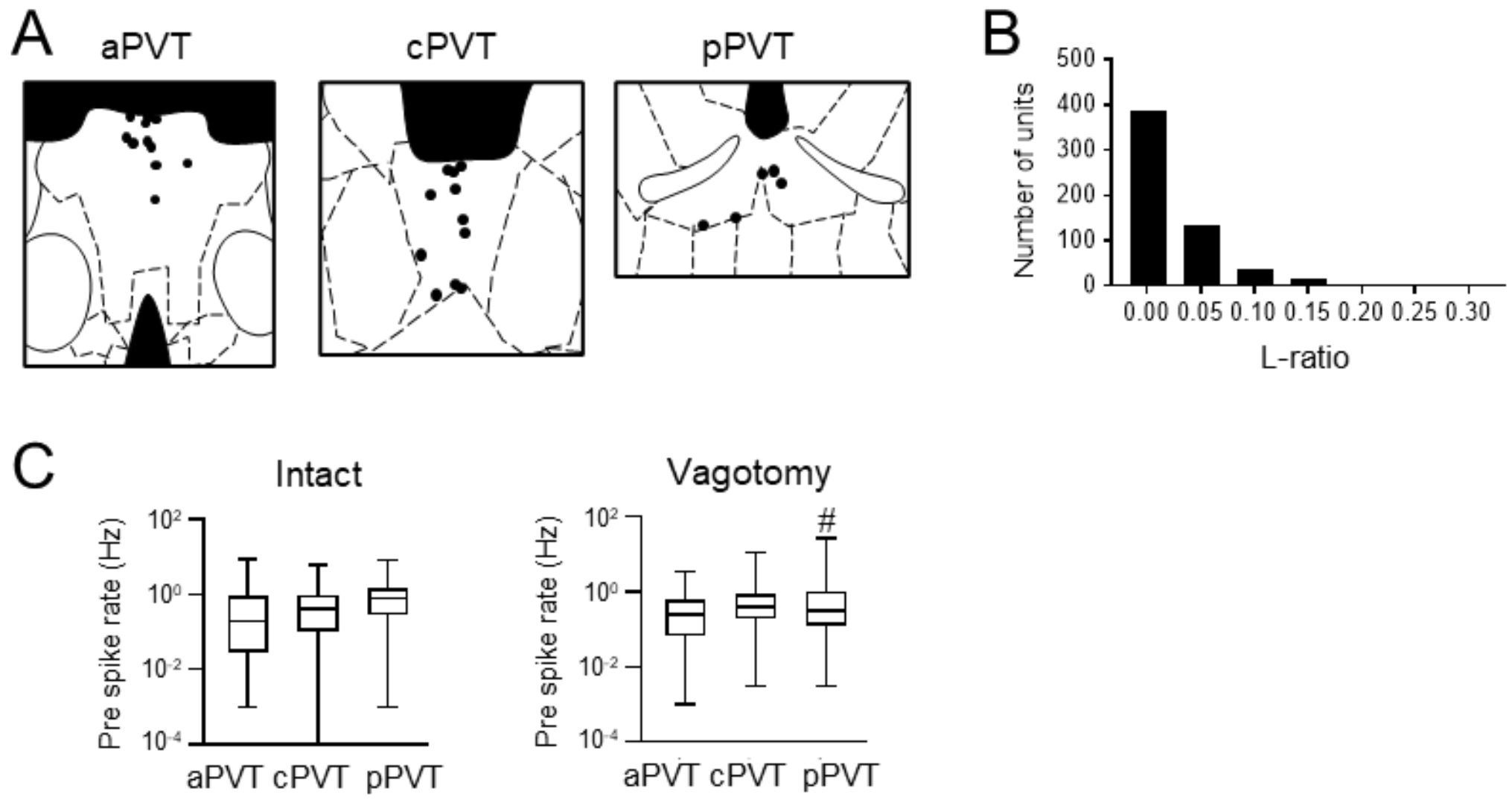

Supplementary Figure 2 Supplementary data for multiunit recording. (A) Summary of electrode locations in each PVT subregion from all mice. Each dot represents each electrode location in PVT subregions. **(B)** Distributions of L_{ratio} of all recorded PVT neurons. **(C)** Baseline (Pre-LPS injection) spike rates of PVT neurons in mice with intact pathway (left, n = 76, 110, and 117 cells from 11, 11, and 5 mice, respectively) and vagotomy (right, n = 80, 123, and 69 cells from 9, 7, and 5 mice, respectively). Intact vs. vagotomy: aPVT, n = 76 and 80 cells from 11 and 9 mice, Z = 0.40, P = 0.69; cPVT, n = 110 and 123 cells from 11 and 7 mice, Z = 0.88, P = 0.38; pPVT, n = 117 and 69 cells from 5 mice each, Z = 2.50, $^{\#}P$ = 0.012; Mann–Whitney U test.

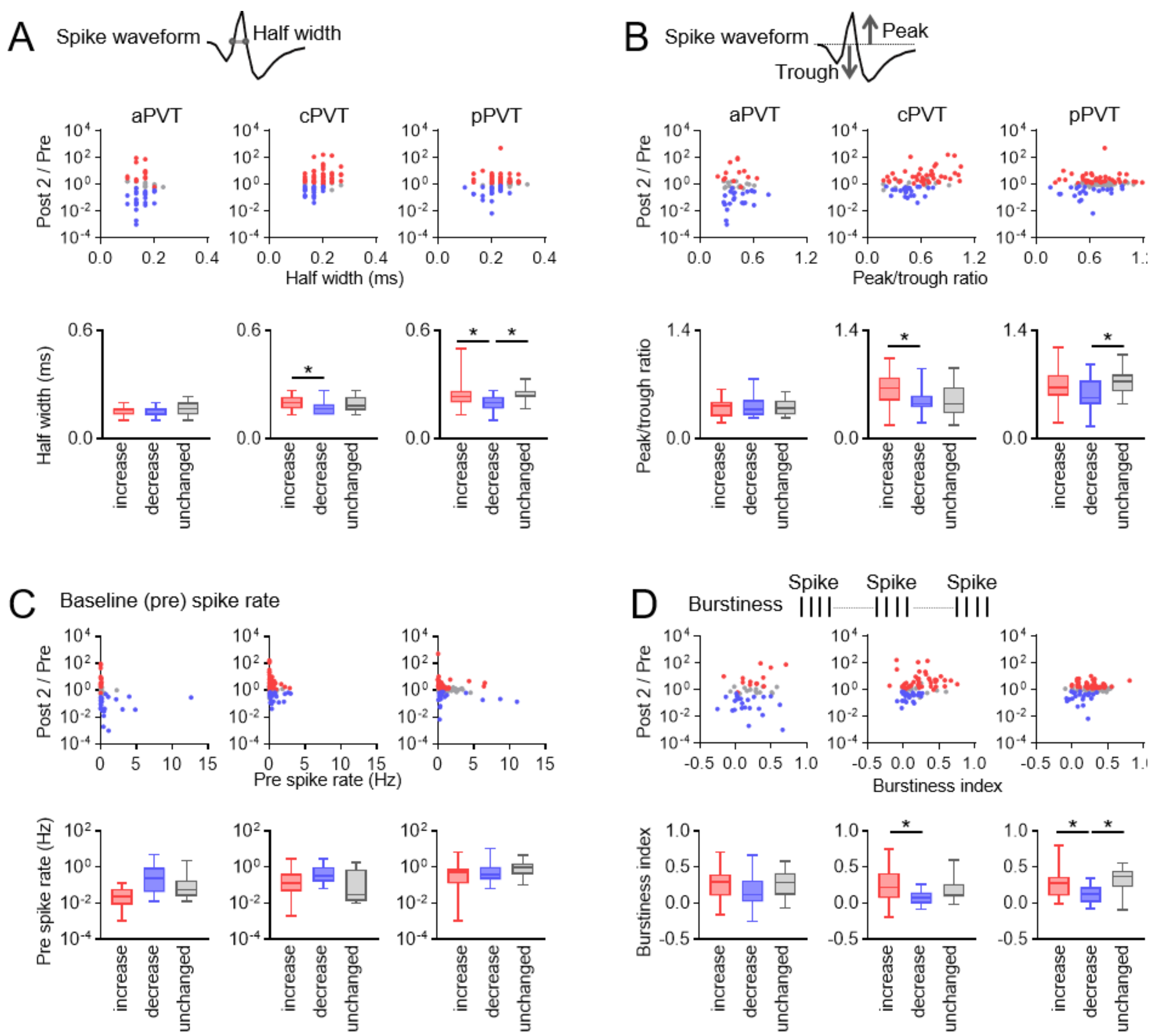

Supplementary Figure 3 Relationship between electrophysiological properties and neuronal responses to VN$_{left}$-independent pathway activation. (A) (Top) Scatter plot showing the relationship between spike half-width and the spike rate change ratio (Post 2/ Pre). Each dot represents an individual PVT neuron (aPVT, *n* = 52 cells; cPVT, *n* = 79 cells; pPVT, *n* = 110 cells). Red, neurons with significantly increased spike rates; blue, neurons with significantly decreased spike rates; gray, neurons with no significant change following LPS injection. (Bottom) Box plots showing spike half-width in neurons with increased (red), decreased (blue), or unchanged (gray) spike rates. cPVT, increase vs. decrease, **P* = 0.0088; pPVT, increase vs. decrease, **P* = 0.0029; unchanged vs. decrease, **P* = 0.0014, Tukey's test. **(B)** Same as (A), but for the peak-to-trough ratio. cPVT, increase vs. decrease, **P* = 0.013; pPVT, unchanged vs. decrease, **P* = 0.0051, Tukey's test. **(C)** Same as (A), but for baseline firing rate. **(D)** Same as (A), but for the burstiness index. cPVT, increase vs. decrease, **P* = 0.0027; pPVT, increase vs. decrease, **P* = 0.0017; unchanged vs. decrease, **P* = 3.6×10^{-6}, Tukey's test.

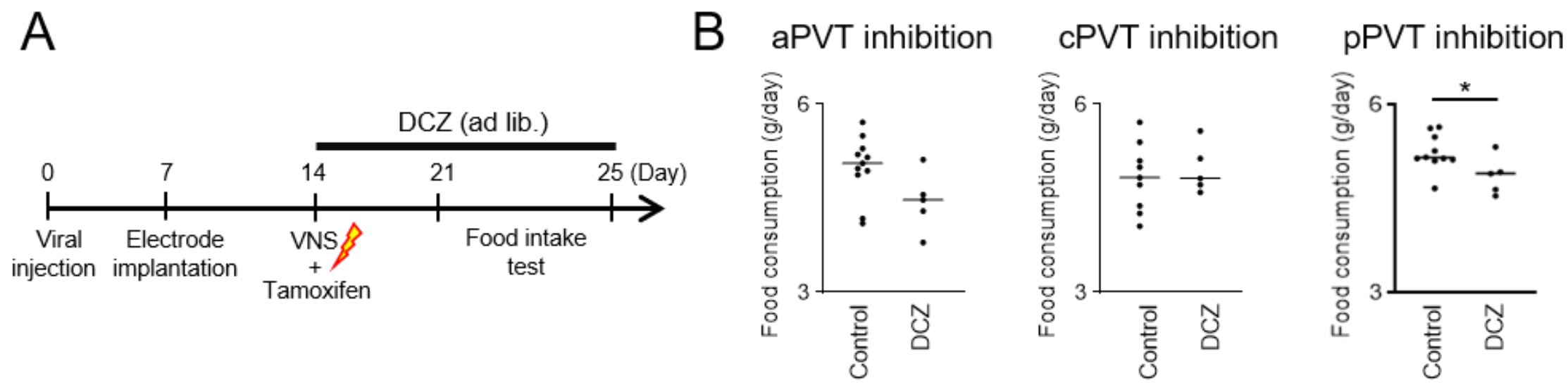

Supplementary Figure 4 Chemogenetic inhibition of VNS-responsive pPVT neurons reduces food consumption. (A) Experimental timeline for food consumption measurement. **(B)** Amount of food consumed during aPVT inhibition (left; n = 11 and 5 mice; t_{13} = 2.09, P = 0.055), cPVT inhibition (middle; n = 9 and 5 mice; t_{12} = 0.51, P = 0.62), and pPVT inhibition (right; n = 10 and 5 mice; t_{13} = 2.28, P = 0.040, Student's t-test).
